# Temperature Compensation of Elasto-Magneto-Electric (EME) Sensors in Cable Force Monitoring Using BP Neural Network

**DOI:** 10.3390/s18072176

**Published:** 2018-07-06

**Authors:** Ru Zhang, Yuanfeng Duan, Yang Zhao, Xuan He

**Affiliations:** 1 Department of Civil Engineering, Zhejiang University City College, Hangzhou 310015, China; zhangru_306@163.com (R.Z.); ceyzhao@zju.edu.cn (Y.Z.); hexuan2010@126.com (X.H.); 2 College of Civil Engineering and Architecture, Zhejiang University, Hangzhou 310058, China

**Keywords:** elasto-magneto-electric (EME) sensor, stress/force monitoring, elasto-magnetic (EM) effect, temperature compensation, back propagation (BP) neural network

## Abstract

Techniques based on the elasto-magnetic (EM) effect have been receiving increasing attention for their significant advantages in cable stress/force monitoring of in-service structures. Variations in ambient temperature affect the magnetic behaviors of steel components, causing errors in the sensor and measurement system results. Therefore, temperature compensation is essential. In this paper, the effect of temperature on the force monitoring of steel cables using smart elasto-magneto-electric (EME) sensors was investigated experimentally. A back propagation (BP) neural network method is proposed to obtain a direct readout of the applied force in the engineering environment, involving less computational complexity. On the basis of the data measured in the experiment, an improved BP neural network model was established. The test result shows that, over a temperature range of approximately −10 °C to 60 °C, the maximum relative error in the force measurement is within ±0.9%. A polynomial fitting method was also implemented for comparison. It is concluded that the method based on a BP neural network can be more reliable, effective and robust, and can be extended to temperature compensation of other similar sensors.

## 1. Introduction

Monitoring of cable stress/force of in-service structures is challenging but crucial to the evaluation of structural safety [1,2]. The elasto-magnetic (EM) effect-based sensors have been receiving increasing attention, with superiorities of noncontact measurement, corrosion resistance, actual-stress measurement, low cost, and long service-life [3,4,5,6]. Owing to the EM effect (also known as the magneto-elastic effect), the action of the stress on the steel member would result in changes in the magnetic properties of the ferromagnetic materials and, thus, in the distribution of the magnetic field in the nearby area. In an elasto-magneto-electric (EME) sensor [6,7], the magneto-electric (ME) sensing unit converts the change of the magnetic field into an easily measured electrical signal represented by voltage, as the function of the ME effect. The sensor system is highly sensitive, has a fast response, and is easy to install.

However, in a sensor system many factors may affect the performance (accuracy and stability) of these sensors in the environment, such as temperature, humidity, power supply fluctuations, noise, and so on. In some cases, uncertain factors that are impossible to predict cause the nonlinearity to vary from instrument to instrument, place to place and time to time. Among these factors, temperature has a major impact on the EME sensor’s performance, which is the focus of this paper. Variation of the ambient temperature affects the magnetic behavior of steel cables, and causes errors in the performance of the sensory system [8,9,10]. The exact mathematical model of the EME sensor showing its dependence on environmental parameters is not available in cable force monitoring [9,10]. Hence, effective measures of compensation should be taken to reduce this temperature disturbance, which should be both efficient and feasible for improving measurement accuracy and reliability.

Compensation methods are divided mainly into hardware and software methods. The hardware compensation method uses symmetric structures, or electronic circuits and components for compensation. For example, Tang et al. devised an EM effect-based sensor with a differential structure using single bypass excitation to compensate for temperature effects in the tension monitoring of steel strands [4,11]. But hardware compensation methods usually suffer from limited measurement accuracy, poor reliability, and poor flexibility owing to manufacturing tolerances, or electronic devices drift, which restrict their applications in engineering practice. Polynomial fitting is one commonly used software compensation method among traditional techniques [3]. It uses a polynomial of degree *n* to approximate a nonlinear curve whose polynomial coefficients can be calculated using experimental data. When new experimental data are obtained, the curve must be refitted, which suggests that this method exhibits poor generalization. Furthermore, it is very difficult to solve the equations when unknown factors are involved. Advanced signal processing techniques and learning algorithms can be employed to make the monitoring system intelligent in terms of error compensation, linearization, and automatic calibration. As a novel information processing method, a BP neural network can be used for temperature compensation of EME sensors owing to their advantages of strong nonlinear mapping ability, parallel processing, error tolerance, adaptive ability, and self-learning capability [12,13,14]. Applications of BP neural network-based approximations with superior performance for mitigating adverse effects of environmental parameters in capacitive pressure sensors [12], prediction of the hot compressive deformation behavior for superalloy nimonic [13], and pattern recognition for the pipeline leakage localization system [14], have been reported.

In this article, the temperature effect on cable force monitoring using an EME sensor was tested and analyzed firstly. The experimental data were also used for the selection of compensation methods. Then, an improved BP neural network was established to compensate for the nonlinear error caused by temperature changes under otherwise randomly varying environmental conditions. Polynomial fitting method was also supplemented for comparison.

## 2. Response of EME Sensors under Different Temperature and Force Conditions

**Figure 1:**
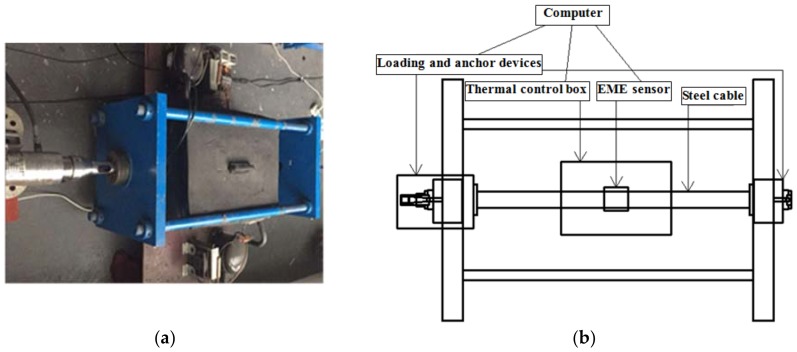
Experimental setup: (**a**) Photo; (**b**) Schematic diagram.

**Figure 2:**
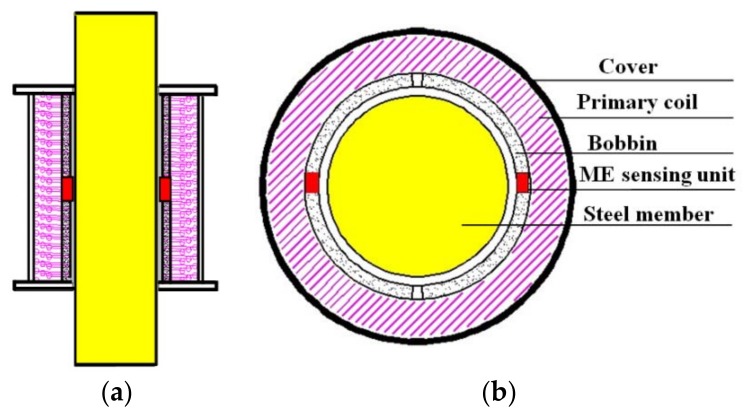
Structure of an EME sensor: (**a**) vertical section view; (**b**) cross-section view.

Experiments were performed to acquire knowledge about the thermal characteristics of the measurement system. The response of EME sensors under different temperature and force conditions should be obtained in advance to select an appropriate compensation method. An experimental platform constructed in our laboratory is shown in Figure 1. The hydraulic prestressing jack (YDC250, Liuzhou OVM Engineering Co. Ltd., Liuzhou, China) was used to stretch the steel cable to the necessary tensile load (force) indicated by a load cell. A thermal control box (450 mm × 400 mm × 260 mm) with an accuracy of ±1 °C was used to provide the constant temperature environment, which partially surrounded the cable. To maintain the desired temperature, it used a resistance heater to heat the sample and Freon to cool down it. The novel EME sensor and measurement system used here were developed by our research team. As illustrated in Figure 2, the manufactured EME sensor is mainly composed of a magnetic excitation part and smart ME sensing units. The magnetic excitation part can be served by the magnetic coil wound on the bobbin. The smart ME sensing unit(s) is/are inserted in the pre-set slot of the bobbin. Their working principle and detailed design information can be found in the previous work [7]. The EME sensor was also placed in the thermostat and mounted at the middle of the steel cable.

**Figure 3:**
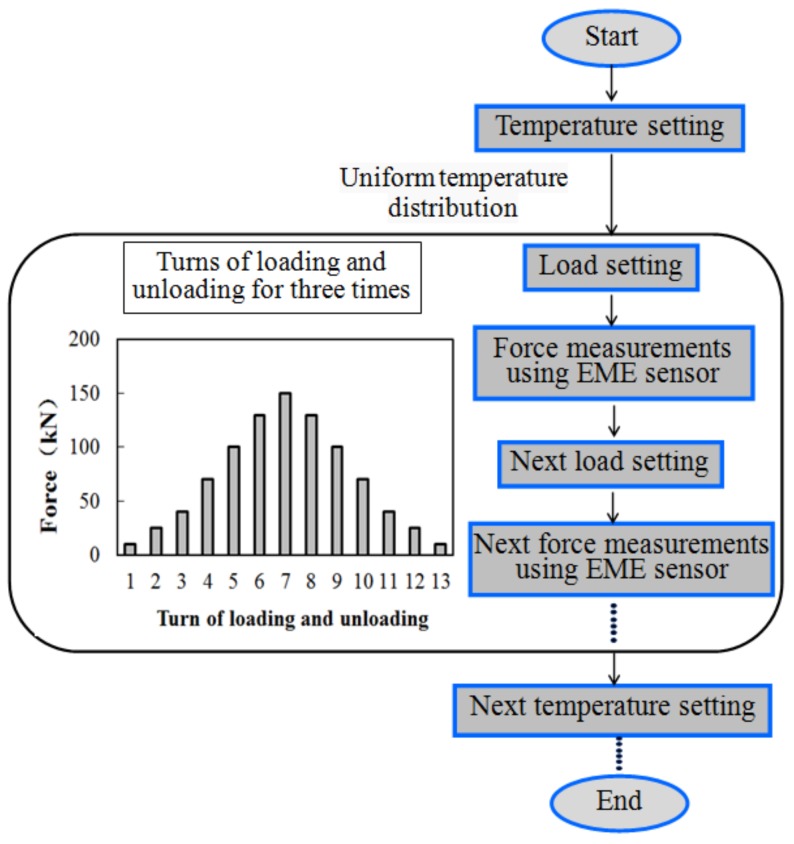
Diagram of the experimental procedure.

In this experiment, the temperature was increased from approximately −10 °C to 60 °C at 10 °C intervals. Figure 3 shows the experimental procedure. When a given temperature was reached, it was maintained for at least 15 min before testing to ensure that the temperature in the box was uniformly distributed. Then the steel cable was pulled step by step with the turns of loading and unloading three times. The maximum load was 150 kN below the yield strength of the steel cable. At each loading level, the force measurements were conducted 11 times for stable magnetization and precise results in this experiment. Then the average output of the EME sensor at each temperature point was calculated and stored. All the input and output was displayed and processed in the computer using the LabVIEW software. The other environment conditions were random with no manual control or sudden changes during this process.

**Figure 4:**
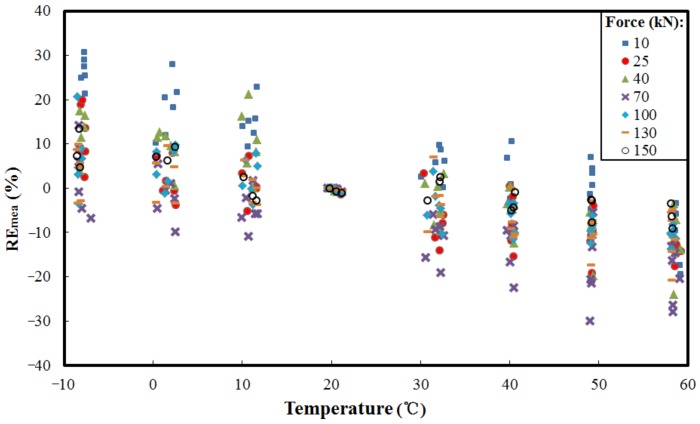
Relative error (${\mathrm{RE}}_{\mathrm{mea}}$ ) of the output voltages from the EME sensor before compensation at different temperatures.

Figure 4 shows the relative error (${\mathrm{RE}}_{\mathrm{mea}}$) of output voltages from the EME sensor before compensation at different temperatures. It can be seen that the output voltage of the EME sensor varies with the different load levels, and drifts owing to temperature fluctuation. At each load level, the measurement error due to temperature variation is computed by reference to the state at room temperature ($T_{0}=20$ °C). Here the ${\mathrm{RE}}_{\mathrm{mea}}$ used is given by

(1) $${\mathrm{RE}}_{\mathrm{mea}}=\frac{V_{T}-\bar{V_{0}}}{\bar{V_{0}}}\times 100\%$$

where $\bar{V_{0}}$ is the mean value of output voltages from EME sensor at temperature $T_{0}$, and $V_{T}$ is the output voltage from the EME sensor at any temperature T.

The maximum |${\mathrm{RE}}_{\mathrm{mea}}$| (absolute value of ${\mathrm{RE}}_{\mathrm{mea}}$) of the sensor’s output is approximately 30.75% before compensation. Several factors contribute to these large error values. First, although the magnetic properties of ferromagnetic materials, such as structural steels, are sufficiently sensitive to stress, they are also influenced by temperature [9,15,16]. Second, mismatched thermal expansion coefficients of packaging materials may have a dramatic effect on the output of sensors if the environmental temperature varies. Furthermore, electric drift and thermal drift occur easily in the electromagnetic system. It is also noticeable that there are slight differences between the loading and unloading results, which have been analyzed in our previous research [17]. As the analysis indicates, the temperature obviously affects the force measurement results. The exact mathematical model of the EME sensor showing the relationship between the measured force and its response, and its dependency on the environmental parameters is not available for our current investigation. Further, as the sensor may exhibit somewhat nonlinear response characteristics, and the environmental parameters influence its behavior nonlinearly, the problem of obtaining an accurate readout and its calibration becomes more complex. To eliminate these influences, it is necessary to find a compensation method.

## 3. Temperature Compensation by Polynomial Fitting Method

Polynomial fitting is widely used to fit models empirically to data, in which case the mathematical function is always available. Here, temperature compensation using polynomial fitting methods based on least-squares fitting [18] is implemented.

The correlation between the temperature and the output of the EME sensor under different forces is first evaluated experimentally. Then, its mathematical expression can be obtained through polynomial fitting as the temperature is changed from −10 °C to 60 °C. The mathematical function is stored in the microprocessor. Thus, the corresponding compensation value for each real-time temperature tested is calculated and subtracted from the actual output of the EME sensor. During construction of the compensation model, special consideration must be given to the precision, practicability, and complexity of the model to satisfy the engineering requirements.

Here, linear and quadratic fitting methods were evaluated respectively. Their mathematical models can be expressed by the following regression equations [18,19]:

(2) $$F_{P1}=a_{0}+a_{1}V_{T}+a_{2}T$$

(3) $$F_{P2}=a_{00}+a_{10}V_{T}+a_{01}T+a_{20}V_{T}{}^{2}+a_{11}V_{T}\cdot T+a_{02}T^{2}$$

where $F_{P1}$ and $F_{P2}$ represent the forces predicted by the linear and quadratic fitting methods, respectively. Further, $a_{0}$, $a_{1}$, and $a_{2}$ are the linear fitting coefficients; $a_{00}$, $a_{10}$, $a_{01}$, $a_{20}$, $a_{11}$, and $a_{02}$ are the quadratic fitting coefficients.

Using the above obtained experimental data, these fitting coefficients can be obtained based on least-squares. In this work, it is completed by using the MATLAB toolbox for polynomial fitting. Then the predicted forces $F_{P1}$ and $F_{P2}$ at any temperature obtained using Equations (2) and (3), respectively. The relative error (RE) and root mean square error (RMSE) between the predicted output and the known applied input are the most common indicators to provide a numerical description of the goodness of the model estimates. They are calculated and defined according to Equations (4) and (5), respectively.

(4) $${\mathrm{RE}}_{i}=\frac{F_{i,P}-F_{i,A}}{F_{i,A}}\times 100\%$$

(5) $$\mathrm{RMSE}=\sqrt{\frac{\sum_{i=1}^{i=N}\left(F_{i,P}-F_{i,A}\right)}{N}}$$

where ${\mathrm{RE}}_{i}$ is the relative error, $F_{i,P}$ is the force predicted by the models (polynomial fitting or BP neural network models), and $F_{i,A}$ is the known applied force for the *i*th data set, respectively. Further, *N* is the number of the predicted data set. RMSE indicates the residual errors, which gives a global idea of the difference between the predicted and applied values. REs of all the data give the error distribution of the models. A smaller absolute value |RE| indicates a more accurate predicted result.

**Figure 5:**
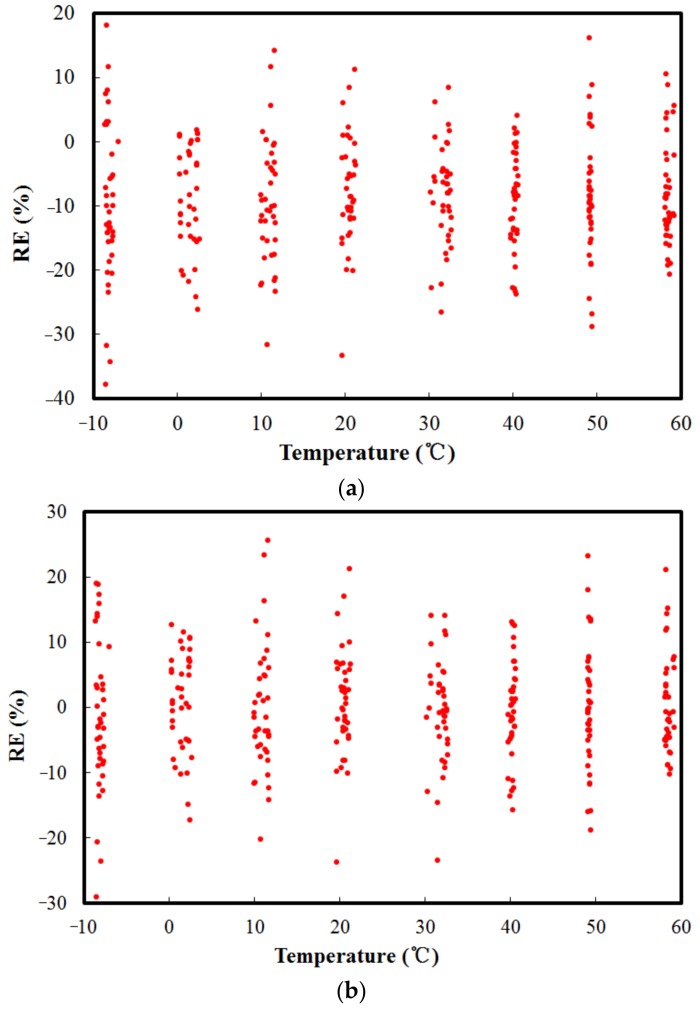
Compensation results obtained using polynomial fitting method: (**a**) RE of applied force and predicted force using linear fitting method; (**b**) RE of applied force and predicted force using quadratic fitting method.

Figure 5a,b shows the compensation results obtained using the linear and quadratic polynomial fitting methods, respectively. It is observed that for the compensated sensor, the maximum |RE| of the measured forces in the temperature range between −10 °C and 60 °C after compensation were 37.98% and 29.11%, with RMSEs of 9.3 and 8.36, respectively. It can be concluded that these polynomial fitting methods are not applicable to this problem.

## 4. Temperature Compensation by BP Neural Network

### 4.1. BP Neural Network Model

**Figure 6:**
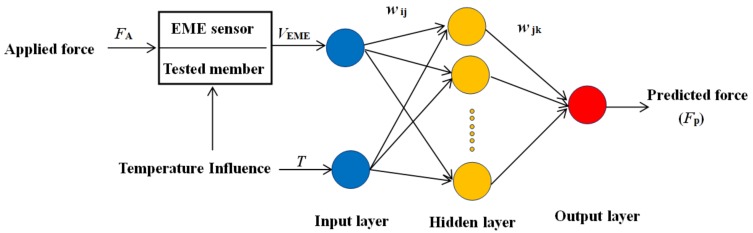
Structure of the BP neural network for compensation.

The structure of the BP neural network for compensation in this modeling is shown in Figure 6, which includes an input layer, a hidden layer, and an output layer. The input layer has two nodes, and the corresponding input variables are the temperature T, and output voltage of the EME sensor $V_{EME}$. The desired (or target) output for the network is the applied force $F_{A}$. The output of the BP neural network is the predicted force $F_{P}$. Further, *w*_ij_ is the weight value connecting the input layer and the hidden layer; *w_jk_* is the weight value connecting the hidden layer and the output layer. Here, *i* = 1, 2; *j* = 1, 2, …, *n*; *k* = 1. To obtain the best generalization performance, it is necessary to optimize the key parameters of the neural network, such as the number *n* of hidden layer neurons, the learning rate, and the training steps, which are performed in the following training process.

### 4.2. Training of BP Neural Network

The training process of BP neural network in this work has the following steps:

(1) Preparation of data sets.

There are 312 data sets obtained from the above experiment, which were used in the above polynomial fitting method. In this modelling, for training the network they are randomly segregated into two parts: 280 data sets are selected as the training set, the rest 32 data sets are used as the testing set. All the parameters used in the simulation study are suitably normalized so as to keep their values within [0, 1].

(2) Network initialization.

The initialized weights (*w_ij_* and *w_jk_*) are set to some random values between 0 and 1. The initialized thresholds of the hidden layers and output layers are fixed to some random values between −1 and 1. The weights and threshold are updated using the BP algorithm. 

The essence of the training process is to iteratively reduce the error between the predicted value and the target (actual) value. The mean-squared error function is used to represent the accuracy of the neural network mapping after a number of training cycles have been implemented. The learning rate [20] may affect the network’s training accuracy and training speed. As the learning rate decreases, the training accuracy increases, at the cost of more training time. However, a too-high learning rate can cause instability in the network, which may fail to converge. There is no integrated theory for determining the learning rate, which is usually set between 0.001 and 1. A learning rate of 0.1 was sufficient to ensure satisfactory training with high accuracy and high training speed in this work. The target training error [20] was set to 0.00001 to ensure comparability.

(3) Selection of optimal architecture and training parameters.

In this step, we train the model using the training set and evaluate it using the validation set. To achieve good results, the BP neural network has to be trained exhaustively with sufficient inputs (training parameters). The well-known BP algorithm is based on an error correction learning rule. The error signal, which is the difference between the actual and desired responses, is back-propagated through the network from the output side to the input side, and the weights in different layers are adjusted to make the actual response approach the desired response. Then the weights are frozen and stored in a read-only memory for testing of the model to verify its performance and for actual use. The simulation studies are conducted using different training parameters. The maximum |RE| is used as a performance criterion. 

**Figure 7:**
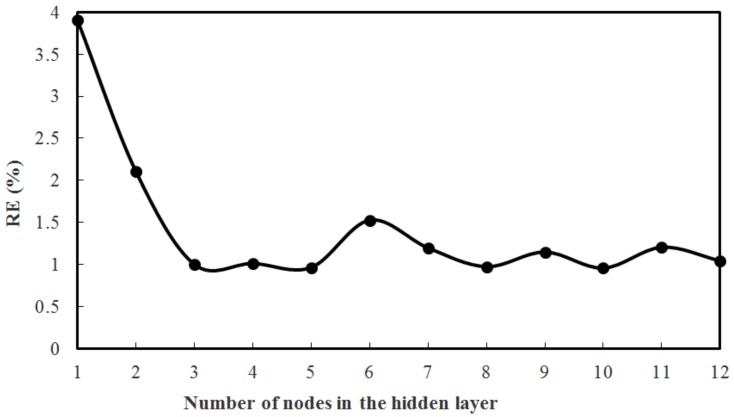
Performance of BP neural network with different numbers of nodes in the hidden layer.

**Figure 8:**
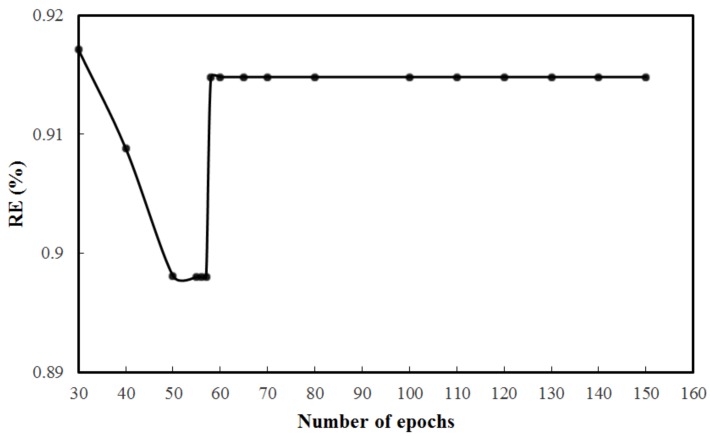
Training of BP neural network.

From Figure 7, it can be seen that the hidden layer with five nodes is optimal in this model, as it has the minimum error |RE| and requires less training time. It is worth mentioned that a neural network with too many nodes in the hidden layer will not only have a longer training time, but also will easily run into the over-training problem. Here a 2-5-1 BP neural network, which is expected to have the best generalization performance, is chosen. The size of the training epoch (defined as one full pass through the training set) affects the general ability of the neural network [20,21]. Too large a size of the epoch will lead to the overfitting of the training set and more convergence time, while an overly small epoch size will impair recognition of the neural network. After each epoch, the order of training patterns will be randomly changed, and the training will continue for the next epoch with the newly arranged patterns. From Figure 8, it is concluded that 70 epochs are sufficient to train this model with effective learning. For fitting and avoiding over fitting, the maximal number of training epochs in this study is set to 100.

Using the same number of nodes in the hidden layer, we then studied the performance of different error minimization algorithms, which correspond to training functions, to identify the most suitable algorithm that yields the lowest |RE|. Table 1 compares the performance of the BP neural network using different training algorithms [21,22]. It can be seen that the gradient descent BP with momentum algorithm, which corresponds to the Traingdm function, has the fastest training speed, whereas its prediction performance is quite bad with the highest |RE|. Furthermore, we can see that the Levenberg-Marquardt algorithm, which corresponds to the Trainlm function, yields the lowest |RE|. Thus, Levenberg-Marquardt algorithm was employed in this research.

The most used transfer functions (also known as activation functions) [23] in the neural network are the log-sigmoid transfer function (Logsig), hyperbolic tangent sigmoid transfer function (Tansig), and linear transfer function (Purelin). Table 2 shows the performance of the BP neural network using these different transfer functions. It was observed that the BP neural network with the Tansig function and the Purelin function as the transfer function of the hidden layer and output layer respectively, was capable of achieving satisfying statistical results with the lowest |RE|. Although other combinations could obtain relatively fast training speeds, their prediction performances were not so good.

### 4.3. Compensation Results

**Figure 9:**
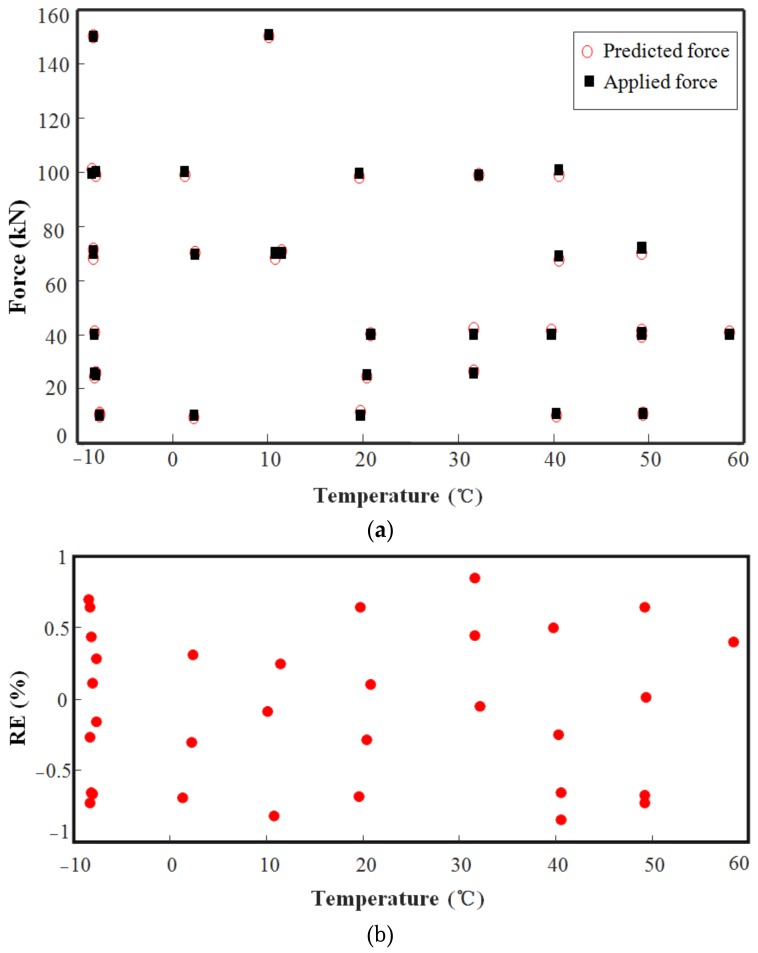
Compensation results obtained using BP neural networks: (**a**) Relationship betweenapplied force and predicted force after compensation at different temperatures; (**b**) RE of applied force and predicted force.

By using the above optimal parameters of the BP neural network, the final compensation results were obtained through training. To assess the compensation effect, some experimental data for model validation which were not used to train the network are selected randomly. Figure 9a compares the test results before and after compensation, namely the applied force output of the load cell and the predicted force output of the EME sensor after compensation. From Figure 9b, we see that for the compensated sensor, the maximum |RE| of the measured forces in the temperature range between −10 °C and 60 °C was less than 0.9% after compensation. In addition, the RMSE has been computed to be approximately 0.39, revealing a reasonable neural network model. Therefore, the BP neural network exhibited good compensation for the temperature error of this sensor, improving the accuracy and stability.

By comparison of Figure 5 and Figure 9, it can be concluded that BP neural network has higher accuracy, a much smaller error, and greater parallel processing capacity to compensate for the temperature error. In particular, under harsh operating environments, polynomial fitting equations have to be reestablished and are usually hard to perform. The BP neural network has the advantages of nonlinear fitting and mode identification capability, regardless of the mathematical model of the sensors and various nonlinear factors. Moreover, it is self-adaptive in constructing a mathematical model after several repeated learning and testing phases. Thus, the advantages of the BP neural network for this type of compensation are notable.

## 5. Conclusions

The performance of EME sensors in cable force monitoring is greatly affected by temperature variations and relevant environmental conditions. In this paper, the response of EME sensors in cable force monitoring under different temperature and force conditions is studied experimentally. It is found that polynomial fitting methods are not applicable to this problem. Then an improved BP neural network model is established to perform this temperature compensation. The test results show that over a temperature range of −10 °C to 60 °C, the maximum error |RE| of the force measurement decreases remarkably, from 30.75% to 0.9% after compensation. And, the computed RMSE of less than 0.4 indicates a reasonable neural network model. Compared with hardware compensation and polynomial fitting methods, the implementation of BP neural network compensation is more reliable, economical, and robust. The smart EME sensor performs quite satisfactorily irrespective of any change in the temperature after compensation. This method can be extended to the temperature compensation of other similar sensors.

## Figures and Tables

**Table 1:** Performance of BP neural network using different training algorithms.

Training Algorithm	Training Function	Training Time (s)	|RE| (%)
Gradient descent BP algorithm	Traingd	5	15.58
Gradient descent BP with momentum algorithm	Traingdm	3	52.37
Gradient descent BP with adaptive learning rate algorithm	Traingda	4	11.28
Gradient descent BP with momentum and adaptive learning rate	Traingdx	4	15.1
Levenberg-Marquardt BP algorithm	Trainlm	7	0.9644
Resilient BP algorithm	Trainrp	4	6.237
Scaled conjugate gradient BP algorithm	Trainscg	4	6.45
Bayesian regulation BP algorithm	Trainbr	7	0.9583
BFGS quasi-Newton BP algorithm	Trainbfg	6	7.093
Cyclic sequential incremental BP algorithm	Trainc	178	6.008

**Table 2:** Performance of BP neural network using different transfer functions.

Transfer Function of Hidden Layer	Transfer Function of Output Layer	Training Time (s)	|RE| (%)
Logsig	Tansig	4	3.756
Logsig	Purelin	4	1.048
Logsig	Logsig	2	39.11
Tansig	Tansig	6	2.471
Tansig	Purelin	7	0.9644
Tansig	Logsig	2	39.11
Purelin	Tansig	2	7.474
Purelin	Purelin	2	4.571
Purelin	Logsig	4	39.11
